# Adolescent obesity, joint pain, and hypermobility

**DOI:** 10.1186/1546-0096-12-11

**Published:** 2014-03-29

**Authors:** Sharon Bout-Tabaku, Sarah B Klieger, Brian H Wrotniak, David D Sherry, Babette S Zemel, Nicolas Stettler

**Affiliations:** 1 Department of Pediatrics, Division of Rheumatology, Nationwide Children’s Hospital, 700 Children’s Drive, Columbus, OH, USA; 2 The Ohio State University College of Medicine, Columbus, USA; 3 Department of Pediatrics, Division of Infection Diseases, The Children’s Hospital of Philadelphia, Philadelphia, USA; 4 Department of Physical Therapy, D’Youville College, Buffalo, USA; 5 Center for Clinical Epidemiology and Biostatistics, University of Pennsylvania School of Medicine, Philadelphia, USA; 6 Department of Pediatrics, Division of Rheumatology, The Children’s Hospital of Philadelphia, Philadelphia, USA; 7 Department of Pediatrics, Division of Gastroenterology and Nutrition, The Children’s Hospital of Philadelphia, Philadelphia, USA; 8 University of Pennsylvania School of Medicine, Philadelphia, USA; 9 The Lewin Group, Falls Church, USA

**Keywords:** Obesity, Adolescent, Puberty, Pain, Joint hypermobility, Lower extremities

## Abstract

**Background:**

Obesity associated with joint pain of the lower extremities is likely due to excessive mechanical load on weight bearing joints. Additional mechanical factors may explain the association between obesity and joint pain.

**Findings:**

We characterized the association between obesity and non-traumatic lower extremity (LE) joint pain in adolescents and examined the modifying effect of hypermobility on this association.

We performed a cross-sectional analysis of data from subjects enrolled in a clinical trial examining the impact of weight loss on bone health in adolescents. Anthropometric data were collected and body mass index (BMI = kg/m^2^) was calculated. Subjects were categorized as obese or healthy weight controls based on CDC 2000 growth curves for age and gender. We assessed any musculoskeletal pain and LE pain by the PEDS™ Pediatric Pain Questionnaire™. Hypermobility was assessed with the modified Beighton scoring system. Multivariate logistic regression models adjusted for covariates were performed to examine the association between weight status and joint pain.

Out of 142 subjects, 91 were obese and 51 were healthy weight. Obesity was not associated with any musculoskeletal pain (OR 0.86, CI 0.49-1.50), LE pain (OR 1.02, CI 0.49-2.15) or hypermobility (OR 1.23, CI 0.72-2.14, p = 0.3). There was no effect modification on the association between obesity and any musculoskeletal pain (OR 0.80, CI 0.45 -1.42) or LE pain (OR 0.98, CI 0.46 - 2.08) by hypermobility status.

**Conclusions:**

We found no association between LE pain and obesity, and hypermobility did not modify this association.

## Introduction

Non- inflammatory joint pain referrals to pediatric rheumatologists are common. The underlying etiologies include trauma, overuse and hypermobility [1-3] With the current obesity epidemic, 16.9 percent of United States (U.S.) children and adolescents are obese and joint pain is more prevalent in obese than healthy weight children [4-6]. One mechanism of obesity related joint pain is that greater load bearing causes micro injury. However little is known about other factors that alter joint loads and possibly cause micro injury among obese children, such as hypermobility.

Hypermobility and obesity together, during rapid growth in puberty, may cause more children to have joint pain and damage, as greater loading stresses are applied to the joint. A single study to date, in British adolescents, found that hypermobility and obesity was a risk factor for musculoskeletal pain [7]. Specifically, the odds ratio of knee pain was 11 in obese, hypermobile subjects compared to 1.57 in non-obese, hypermobile subjects, suggesting that two factors affecting load on the lower extremity joints may be markers for joint stress [7].

We characterized the association of obesity and non-traumatic lower extremity (LE) joint pain in US adolescents and evaluated the modifying effect of hypermobility on this association. We hypothesized that obese adolescents would have a greater prevalence of LE pain compared to healthy weight peers, and that hypermobile, obese adolescents would have a greater prevalence of LE pain than non-hypermobile, obese adolescents.

## Findings

### Subjects

Analysis for this sub study was based on baseline data from obese subjects and a healthy weight control (HW) group enrolled in a randomized clinical control trial examining the impact of weight loss, on bone health in adolescents between January 2008 and August 2011. The study was a nested case–control powered on detecting differences in bone strength between obese and HW subjects required 51 HW subjects. The number of obese subjects was determined based on power analysis to detect change in bone strength between obese subjects in the RCT phase who received standard care or comprehensive weight control.

Subjects were recruited using flyers and radio announcements from the community. They were invited to participate based on inclusion and exclusion criteria. Subjects were excluded for syndromic or secondary obesity, developmental delay, significant psychological or psychiatric disorder, diabetes, any chronic disease or medication interfering with bone health or weight loss, inability of parents to participate in the study visits, cigarette smoking, or recent significant weight loss. All the participants who participated in the RCT had data collected for this sub study at baseline. Baseline visits included the collection of self-reported pain and hypermobility data in addition to the multiple measures related to the primary outcomes of the study of bone health and weight loss. The RCT and the sub study received approval from the Children’s Hospital of Philadelphia institutional review board and all subjects signed informed consents.

### Anthropometric measures

Anthropometric data were collected at the study visit per study protocol. Height was measured using a stadiometer (Holtain, Crymych, UK) to the nearest 0.1 centimeter. Weight was measured on a digital electronic scale (Seca, Munich, Germany) to the nearest 0.1 kilogram. Body mass index (BMI = kg/m2) was calculated and obesity status determined using US CDC 2000 growth charts. Obesity was defined as having a BMI ≥ 95th percentile for sex and age. BMI Z-scores were generated similarly [8]. Younger obese adolescents and slightly older adolescent HW subjects were recruited to account for the advanced maturation expected in the obese subjects [9].

### Measurement of pain

The PEDS™ Pediatric Pain Questionnaire™ assessed present pain and worst pain over the past week [10]. A body map localized areas of musculoskeletal pain across 14 areas including joints. We analyzed pain data that was reported by the adolescent [11]. Anyone answering “yes” to pain at a specific body part was considered to have pain. Adolescents with trauma related pain, headaches and stomach pain were categorized as no pain. Then two pain variables were generated to characterize pain 1) any musculoskeletal pain (including all extremities, neck and back) and 2) pain only in the LE (including the hips, legs, thighs, knees or ankles). This allowed us to separate the effects of weight bearing associated pain vs. pain unrelated to weight bearing.

### Hypermobility measures

Hypermobility measurements were performed by a trained pediatric rheumatologist (SBT) and trained examiners (SK, SN) using the modified Beighton nine-point scoring system [12]. We defined hypermobility two ways: ≥4 hypermobile joints and a more stringent definition, ≥ 6 hypermobile joints [13]. We assessed the popliteal angle to measure hamstring flexibility with a goniometer according to standard technique [14,15]. The inter-observer correlation coefficient of two measurers on a total of 129 subjects was 0.92 and 0.90 for the right and left popliteal angles, respectively.

### Other measurements

Sex, age, race, Tanner stage and physical activity were covariates. Tanner stage was self-reported using a validated questionnaire [16]. Physical activity was assessed using the Actitrac activity monitor (IM Systems, Baltimore, MD), a two-dimensional accelerometer worn on the belt for seven days. The average number of activity counts per minute (cpm) was used as a measure of activity and as a continuous variable in the analysis.

### Statistical analysis

Categorical variables were reported as percentages, and continuous variables as means and standard deviations. Between group differences in pain and hypermobility were tested with chi-square or Fisher’s exact test, and t-tests for normally distributed variables, or Kruskal-Wallis tests for non-normal distributions. The relationship between obesity and LE pain and obesity and hypermobility, were examined using multivariate logistic regression models. A separate model assessed whether the association between obesity and joint pain is modified by hypermobility. A two-tailed p-value <0.05 was considered statistically significant. Stata12.0. (StataCorp) was used for the analyses.

### Characteristics of the cohort

Data were obtained on 142 subjects (91 were obese and 51 were healthy weight). The demographic characteristics of the subjects are shown in Table 1. The HW subjects were older than their obese counterparts by design (14.5 vs. 12.2 years, p < 0.001). As expected, the BMI and BMI-Z-score differed between groups.

**Table 1:** Characteristics of obese subjects and healthy controls

**Variable**	**Obese (n = 91)**	**Controls (n = 51)**	**p value**
Age, years (median)	12.2	14.5	<0.001
Range	10.2 to 17.6	10.2 to 17.9	
Sex, % male	35.2	37.3	0.80
Race, % black	64.8	64.7	0.99
% Tanner 4 or 5	47.7	70.6	0.055
Physical activity (mean cpm, sd)*	272.8 ± 108.2	255.6 ± 92.3	0.38
BMI (mean)	33.9 ± 4.90	19.70 ± 2.00	<0.001
Range	26.3 to 49.6	15.1 to 23.2	
BMI-Z- score (mean)	2.38 ± 0.23	- 0.02 ± 0.62	<0.001
Range	1.93 to 2.88	-1.54 to 0.99	

Statistical analysis for continuous variables was performed using t-tests for normally distributed variables. For categorical variables, the Chi 2 test was used. SD = standard deviation.

Physical activity is in counts per minute (cpm) of physical activity.

*Physical activity data were available on 125 subject (obese = 82, non obese = 43).

Body Mass index (BMI).

BMI-Z-score adjusted for age and gender based on US 2000 CDC growth charts.

### Obesity, hypermobility and joint pain

LE pain prevalence was 14% among obese and 10% among HW subjects; but obesity was not associated with any musculoskeletal pain (OR 0.96, CI 0.64-1.44, p = 0.8) or LE pain (OR 1.29, CI 0.75-2.23, p = 0.3) in unadjusted and adjusted models (Table 2). Hypermobility (defined as ≥ to 4 joints) prevalence was 14% among obese, and 9% among HW subjects. With the stringent definition, (hypermobility ≥ 6 joints), the prevalence was 2% for both groups. (Table 3) Obesity was not associated with hypermobility (OR 1.23, CI 0.72-2.14, p = 0.3 in unadjusted and adjusted models. (Table 4) Hypermobility did not differ by sex or race.

**Table 2:** Association of pain with BMI Z-score and obesity

		**1. Unadjusted**		**2. Adjusted**	
**Pain**	**Covariate**	**OR**	**95% CI**	**OR**	**95% CI**
Musculoskeletal	**BMI-Z-score**	0.99	0.72 - 1.36	1.01	0.64 - 1.58
Lower Extremity	**BMI-Z-score**	1.10	0.73 - 1.66	0.98	0.56 - 1.72
Musculoskeletal	**Obesity**	0.96	0.64 - 1.44	0.86	0.49 - 1.50
Lower Extremity	**Obesity**	1.29	0.75 - 2.33	1.02	0.49 - 2.15

Adjusted for sex, age, race, Tanner stage, hypermobility and physical activity.

Odds ratios (OR’s) > 1 indicate increased odds of having any musculoskeletal pain or lower extremity pain. OR’s <1 indicate a decreased odds of having any musculoskeletal pain or lower extremity pain. Results are considered statistically significant if the 95% Confidence interval (CI) does not include 1.

**Table 3:** Pain and hypermobility

	**Obese (n = 91)**	**Non-obese (n = 51)**	**p value**
***Musculoskeletal pain***			
Any musculoskeletal pain (%)	22	24	0.83
Lower extremity pain (%)	14	10	0.31
***Hypermobility***			
Hypermobile 6 (%)	2	2	0.71
(≥6 Beighton score)			
Hypermobile 4 (%)	14	10	0.31
(≥4 on Beighton score)			
Thumbs (%)	24	25	0.86
Elbows (%)	8	22	0.02
Knees (%)	13	12	0.81
Fingers (%)	9	2	0.10
Trunk (%)	4	2	0.41

All values are percentages. For categorical variables, the Chi 2 or the Fisher’s exact test was used where appropriate.

**Table 4:** Association of hypermobility with BMI Z-score and obesity

		**1. Unadjusted**		**2. Adjusted**	
	**Covariate**	**OR**	**95% CI**	**OR**	**95% CI**
Hypermobility4*	**BMI-Z-score**	1.12	0.74 -1.72	0.72	0.42- 1.22
Hypermobility4	**Obesity**	1.23	0.72 – 2.14	0.79	0.39- 1.60

Adjusted for sex, age. Race, Tanner stage, and physical activity.

*Hypermobility4 is the less stringent level of the Beighton score (4/9).

Odds ratios (OR’s) > 1 indicate increased odds of having hypermobility. OR’s <1 indicate a decreased odds of having hypermobility. Results are considered statistically significant if the 95% Confidence interval (CI) does not include 1.

The mean right popliteal angles were 136° and 119°, for the obese and HW subjects, respectively. The mean left popliteal angles were 136° and 120° for the obese and HW subjects, respectively. The standard deviation was 15°. These angles differed by obesity status with a p < 0.001.

There was no effect modification by hypermobility status, on the association between obesity and LE pain 0.98 (95% CI = 0.46 - 2.08) or musculoskeletal pain 0.80 (95% CI = 0.45 -1.42) (Table 5).

**Table 5:** Association of hypermobility and pain

		**1. Unadjusted**		**2. Adjusted**	
	**Covariate**	**OR**	**95% CI**	**OR**	**95% CI**
MSK pain	**Hypermobility4**	1.88	0.65 - 5.50	2.61	0.76 - 8.89
LE pain	**Hypermobility4**	1.35	0.35 – 5.19	1.46	0.32 - 6.55
MSK pain	**Hypermobility4x**			0.80	0.45 - 1.42
	**Obesity§**				
LE pain	**Hypermobility4x**			0.98	0.46 – 2.08
	**Obesity§**				

Adjusted for sex, age, race, Tanner stage, obesity and physical activity.

Odds ratios (OR’s) > 1 indicate increased odds of having MSK pain or Le pain. OR’s <1 indicate a decreased odds of having MSK pain or LE pain. Results are considered statistically significant if the 95% Confidence interval (CI) does not include 1.

**§**Hypermobility4xObesity is the interaction term in the model looking at the association of obesity and joint pain.

## Discussion

We are the first to examine the associations between obesity, hypermobility and non-traumatic LE pain in US adolescents. Obese adolescents compared to healthy weight subjects did not have a greater prevalence of LE pain or hypermobility. We found that the combination of obesity and hypermobility did not confer greater odds of reporting LE joint pain.

Consistent with our findings of 14%, the prevalence of LE joint pain in obese adolescents ranges from 12- 44% [5,17-19]. A recent study in 5000 British 17 year olds showed an association between obesity and knee pain (OR 1.81 CI 1.27–2.74) [6]. Although this was a large study the population was racially homogenous and limited to only 17 year olds. Our data, with fewer subjects and greater racial heterogeneity, showed a greater percent of adolescents with LE pain were obese but there was no statistical difference from the HW subjects. In a post hoc analysis assuming the prevalence of LE between the two groups (14% vs. 10%), at a significance level of 0.05 and power of 80%, we would have needed a total sample size of 2210 subjects.

The combination of hypermobility and obesity, two pathways associated with joint instability and micro trauma, is a plausible mechanistic hypothesis for why more obese subjects have lower extremity joint pain than non-obese ones [20]. Tobias et al. found that knee pain was prevalent among 17 year olds if at 14 years, they were hypermobile and obese compared to those at 14 years who were hypermobile and non-obese [7]. However, they did not exclude children with injuries, thus the positive association and interaction between obesity and hypermobility on pain could be due to injury instead. Our data excluded children with injuries and we found no evidence to suggest that hypermobility, together with obesity, was associated with LE non-traumatic joint pain. Our findings may differ due to the racial heterogeneity and younger age of our subjects. Our study had more black subjects, who have a lower prevalence of hypermobility than whites, which may account for the lack of association in our group [21]. It is possible that obesity and hypermobility manifests with pain in older adolescents rather than younger ones, as adolescents have completed their bone and peri-articular tissue growth, while younger adolescents may be more resilient and heal from micro injuries.

Popliteal angles were measured to assess hamstring tightness, which has been associated with knee and back pain. Typically as children get older the popliteal angle decreases indicating less hamstring flexibility. Interestingly among our subjects the obese children had larger popliteal angles than the HW ones, indicating more hamstring flexibility which differs from de Sa Pinto et al., who found no difference between obese and HW subjects [19]. As the prevalence of both knee and back pain were low in our study we could not determine how hamstring flexibility among obese children relates to knee or back pain.

Our major limitation is our small sample size, since we were not powered to study obesity and joint pain. In particular the prevalence of hypermobility was low in our subjects, and larger numbers would be necessary to test associations as has been the case in the larger cross-sectional studies [13]. Another limitation was that we did not assess pain intensity since our focus was pain prevalence. Finally, our subjects may not be representative of the general population as they agreed to join an RCT and are possibly more motivated due to greater obesity and more obesity related problems, such as pain.

Our findings add to the sparse evidence about obesity, hypermobility and LE pain in U.S. adolescents. Furthermore, the clinical significance of a difference of 4% greater prevalence of LE pain in obese children may be important, as pain likely limits their interest, function and ability to participate in weight loss programs involving physical activities [22]. Prospective studies with sufficient power and sampling children at different developmental stages are needed to understand how obesity mediated altered mechanics affects the function and structure of the LE joints.

## Abbreviations

LE: Lower extremity; BMI: Body mass index (BMI = kg/m2).

## Competing interest

The authors have no conflicts of interest to disclose.

## Authors’ contributions

SBT was involved in study conception and design, data collection, data analysis and drafting and editing. SK was involved in data collection, data analysis, drafting and editing. BW was involved in data collection, data analysis and editing. DS was involved in study conception and design and editing. BZ was involved in study conception, design and implementation of the clinical trial, and editing. NS was involved in study conception, design and implementation of the clinical trial and the current study. He participated in the data interpretation and editing. All authors were involved in writing the paper and had final approval of the submitted and published versions.
